# Improving Compliance With the World Health Organization Surgical Safety Checklist Through a Clinical Audit at Prince Osman Digna Hospital, Sudan

**DOI:** 10.7759/cureus.104793

**Published:** 2026-03-06

**Authors:** Elmustafa Alkhalifa, Tartel Ahmed, Shima Mohamed Abdallah Alradi, Ward Awadelkarim Elsayed Elhag, Gihad Abdelmonim Mohamed Salih, Rania Ali Eissa Wadi, Abdelsalam Algray Abdelsalam Algray, Afrah Mohamed Idrisshaikh Mohamed, Solima Mohammed Abdelgadir Mohammed, Muhammad Altahir Ahmed Omer, Nidal Youseef AlTaher Aboh

**Affiliations:** 1 Department of Emergency Medicine, Health Service Executive, Dublin, IRL; 2 Department of Surgery, Sudan Medical Council, Khartoum, SDN; 3 Department of General Practice, Ahfad University for Women, Omdurman, SDN; 4 Department of General Practice, Port Sudan Teaching Hospital, Port Sudan, SDN; 5 Department of Surgery, Prince Osman Digna Hospital, Port Sudan, SDN; 6 College of Medicine and Surgery, Karary University, Omdurman, SDN; 7 Department of General Practice, University of Gezira, Wad Madani, SDN

**Keywords:** low-resource settings, operating room, patient safety, perioperative safety, sudan, surgical documentation, surgical safety checklist, world health organization

## Abstract

Background and aim

The World Health Organization (WHO) Surgical Safety Checklist (SSC) is widely used to support safe perioperative practice; however, achieving consistent and complete use in routine clinical settings remains challenging, particularly in resource-limited environments. Quality improvement audits provide a structured approach for evaluating current practice, identifying gaps, and supporting improvements in perioperative processes and team communication. This study aimed to assess baseline compliance with the WHO SSC and evaluate the impact of targeted interventions on checklist adherence at Prince Osman Digna Hospital, Sudan.

Methods

A closed-loop clinical audit was conducted between March and August 2025. The first cycle involved a retrospective review of all surgical cases (n = 101) performed over a two-week period, representing total coverage of eligible procedures. This was followed by a two-month intervention comprising staff education, standardized checklist documentation, and regular feedback. A second prospective audit cycle assessed all consecutive surgical cases (n = 203) performed over a three-month period, again using total coverage sampling. Compliance with SSC components across the Sign In, Time Out, and Sign Out phases was compared between cycles.

Results

Baseline compliance varied widely, with particularly low adherence in communication-dependent checklist items. In the first cycle, team introductions were documented in 22 (21.8%) cases, discussion of anticipated critical steps in 18 (17.8%), and completion of surgical counts in 41 (40.6%). Following the intervention, substantial improvements were observed across most parameters, including team introductions (86 (42.4%) vs. 22 (21.8%), p < 0.001), discussion of anticipated critical steps (66 (32.5%) vs. 18 (17.8%), p = 0.006), surgical counts (132 (65.0%) vs. 41 (40.6%), p < 0.001), specimen labeling (96 (47.3%) vs. 28 (27.7%), p = 0.001), and nursing sterility confirmation (184 (90.6%) vs. 25 (24.8%), p < 0.001). Several domains demonstrated large absolute improvements, while a few items, such as surgical site marking (161 (79.3%) vs. 83 (82.2%), p = 0.554), showed minimal change.

Conclusions

Targeted, low-cost audit interventions significantly improved adherence to the WHO SSC and strengthened perioperative communication. This project demonstrates that audit-driven strategies can effectively enhance surgical safety practices in resource-limited settings and provides a scalable model for ongoing quality improvement.

## Introduction

Patient safety and quality assurance are essential components of contemporary surgical practice, supported by globally recognized standards designed to reduce preventable harm and improve clinical outcomes. The Royal College of Surgeons of England (RCS), in Good Surgical Practice (2014) and its revised 2025 edition, has delineated explicit expectations for safe perioperative care, emphasizing effective communication, multidisciplinary collaboration, precise documentation, and rigorous compliance with standardized safety protocols [1,2]. These principles align closely with the General Medical Council’s Good Medical Practice, which outlines the professional, ethical, and legal responsibilities guiding clinical decision-making and patient care in surgical settings.

The establishment of the World Health Organization (WHO) Surgical Safety Checklist (SSC) in 2009, as part of the Safe Surgery Saves Lives campaign, marked a major advance in global surgical safety. The checklist was developed to address critical safety concerns during three specific phases of surgery: Sign In, conducted before anesthetic induction; Time Out, performed immediately prior to skin incision; and Sign Out, completed before the patient leaves the operating room. Each phase emphasizes essential safety verifications, including patient identification, procedure and site confirmation, anticipation of key events, and promotion of shared situational awareness within the surgical team. Extensive global evidence demonstrates that consistent application of the SSC is associated with significant reductions in surgical complications, preventable errors, and perioperative mortality [3,4].

The WHO SSC is widely recognized as one of the most effective tools for improving surgical safety worldwide. By integrating systematic safety checks into routine procedures, the checklist enhances communication, accountability, and team coordination. Despite this, implementing the checklist consistently remains challenging in low-resource healthcare settings. In countries such as Sudan, barriers including limited access to standardized documentation, high workload demands, frequent staff turnover, and irregular formal training have been shown to impede effective checklist utilization. As a result, adherence to SSC protocols and associated documentation is often suboptimal, exposing patients to preventable risks and compromising clinical governance and medico-legal safeguards [5].

Evidence from low- and middle-income countries suggests that audit-driven quality improvement initiatives can substantially address these challenges. Clinical audits in tertiary hospitals in Lahore, Pakistan, and Bangalore, India, demonstrated that structured feedback, staff education, and tailored interventions significantly improved adherence to RCS standards and perioperative documentation [6,7]. Similar outcomes have been reported in Sudan, where audit cycles and focused training programs at Dongola and Elobeid Teaching Hospitals led to marked improvements in checklist completion and documentation quality [8-11]. These findings underscore the effectiveness of audit and feedback as pragmatic, low-cost strategies for enhancing patient safety in resource-limited environments.

In July 2025, a quality improvement project was launched at Prince Osman Digna Hospital in Port Sudan, Sudan, informed by this growing body of evidence. Initial assessments revealed inconsistent application of the WHO SSC, lack of standardized operative documentation, and variability in safety practices among surgical teams. Prince Osman Digna Hospital, a regional referral center serving a predominantly rural population with limited healthcare resources, represents a relevant context for evaluating and improving surgical safety protocols. This study aimed to systematically assess baseline adherence to the WHO SSC and implement targeted, audit-informed interventions to enhance compliance and documentation quality, thereby improving surgical outcomes and promoting sustainable quality improvement within the Sudanese healthcare system.

## Materials and methods

Study design and setting

This quality improvement project was conducted at Prince Osman Digna Hospital, a public tertiary referral hospital in Port Sudan, Sudan, using a closed-loop clinical audit design aimed at improving compliance with and documentation of the WHO SSC. The project was implemented over three consecutive phases between March and August 2025, comprising a retrospective baseline audit (Audit Cycle 1), a structured intervention period, and a prospective re-audit (Audit Cycle 2).

Audit standard

The audit standard was based on the WHO SSC, as outlined in the WHO Guidelines for Safe Surgery and the accompanying Implementation Manual [3]. The SSC consists of three mandatory phases: Sign In (before induction of anesthesia), Time Out (before skin incision), and Sign Out (before the patient leaves the operating room). These phases are designed to ensure correct patient identification, verification of the surgical procedure and site, effective multidisciplinary communication, and anticipation of critical perioperative events. Complete and accurate documentation of all checklist components for each surgical procedure was defined as the benchmark standard.

Baseline assessment (Audit Cycle 1 - retrospective)

The baseline audit was conducted retrospectively over a two-week period in March 2025. During this phase, all general surgery operative records (n = 101) from the audit period were reviewed, representing total coverage sampling. Only cases with available operative documentation were included to ensure an accurate representation of routine clinical practice.

The general surgery department was selected because it accounts for the largest surgical workload in the hospital and serves as a major service and training unit. Each operative record was assessed using a standardized data collection tool aligned with WHO SSC requirements. Evaluated parameters included patient identifiers, confirmation of procedure and surgical site, documentation of anesthetic safety checks, anticipated critical events, team member identification and signatures, intraoperative confirmations, and postoperative instructions.

The baseline audit revealed several deficiencies in SSC documentation, particularly during the Sign In and Sign Out phases. Common gaps included incomplete pre-anesthetic verification, absence of documentation of anticipated critical events, lack of team sign-off, and inconsistent recording of postoperative management plans.

Root cause analysis

Following completion of Audit Cycle 1, a structured root cause analysis was conducted to identify factors contributing to incomplete or inconsistent SSC documentation. Key contributing factors included limited awareness of the full scope of WHO and Royal College of Surgeons surgical safety standards, absence of standardized and user-friendly documentation templates, high operative workload with associated time constraints, and lack of routine feedback on documentation quality. Inconsistent induction and training of newly appointed staff were also identified as barriers to sustained compliance.

Intervention phase

Based on the findings of the baseline audit, a two-month intervention period was implemented between April and May 2025. The intervention aimed to reinforce consistent and complete application of the WHO SSC across all surgical procedures through education, supervision, and team accountability.

The interventions were applied uniformly to both elective and emergency surgeries to ensure adherence to safety practices regardless of case urgency. Standardized operative documentation templates were introduced to promote consistency and completeness of records. These templates were specifically designed to address SSC components that were frequently under-documented during Audit Cycle 1 and were fully aligned with WHO SSC requirements.

The core structure of the WHO SSC was maintained in its entirety, with no items omitted. Additional context-specific fields, such as patient address, consultant surgeon name, and postoperative management plan, were incorporated to address locally identified documentation gaps.

A structured educational program was delivered to all members of the surgical team, including surgeons, anesthesiologists, and operating room nursing staff. This program included two interactive workshops and a focused bedside teaching session, emphasizing the rationale for checklist use, effective multidisciplinary communication, and accurate documentation practices. Participants’ understanding was assessed through immediate post-training quizzes and supervised checklist simulations. Visual reminders, including printed checklists and posters, were displayed prominently within operating rooms, and senior clinicians provided ongoing supervision and feedback.

Post-intervention assessment (Audit Cycle 2 - prospective)

The re-audit was conducted prospectively over a three-month period, from June to August 2025. During this phase, 203 consecutive operative cases were assessed in real time following full implementation of the intervention. Both elective and emergency procedures were included.

The same data collection tool and assessment criteria used in Audit Cycle 1 were applied to ensure methodological consistency and allow valid comparisons between audit cycles. Each operative record was evaluated for completeness, clarity, and accuracy of WHO SSC documentation using the standardized templates introduced during the intervention phase.

The larger sample size in Audit Cycle 2 reflected improved documentation practices, enhanced staff engagement, and broader case capture following sustained reinforcement of checklist use.

Data analysis

Data were analyzed using descriptive and inferential statistical methods. Compliance with individual SSC parameters was summarized as frequencies and percentages for both audit cycles. Comparative analysis was performed to assess changes in compliance between Audit Cycle 1 (n = 101) and Audit Cycle 2 (n = 203). Where appropriate, the chi-square test was used to evaluate the statistical significance of differences between cycles, with p-values < 0.05 considered statistically significant.

Ethical considerations

Ethical approval for this quality improvement project was obtained from the Prince Osman Digna Hospital Research and Ethics Committee and hospital administration. As the project was classified as a local clinical audit aimed at service improvement, a formal institutional review board reference number was not issued. The audit involved review of operative documentation only and did not include patient identifiers. Confidentiality and data privacy were strictly maintained, with all data anonymized prior to analysis.

The project was conducted in accordance with the principles of the Declaration of Helsinki, with the primary aim of improving patient safety and quality of surgical care and without exposing patients to additional risk.

## Results

A total of 304 surgical procedures were assessed across the two audit cycles, including 101 cases in Cycle 1 and 203 cases in Cycle 2. Overall compliance with multiple WHO SSC items improved in Cycle 2, with the largest gains observed during the Time Out and Sign Out phases.

In the Before Induction of Anesthesia domain, confirmation of patient identity, surgical site, procedure, and consent remained high, increasing from 89 (88.1%) in Cycle 1 to 186 (91.6%) in Cycle 2 (p = 0.327). Surgical site marking showed a small, non-significant decline from 83 (82.2%) to 161 (79.3%) (p = 0.554). Anesthesia machine and medication checks remained consistently high, recorded in 100 (99.0%) versus 200 (98.5%) cases (p = 0.725), while pulse oximeter use increased slightly from 98 (97.0%) to 199 (98.0%) (p = 0.620). Documentation of known allergies improved from 16 (15.8%) to 45 (22.2%) (p = 0.186). Assessment of blood loss risk greater than 500 mL increased from 10 (9.9%) to 35 (17.4%) (p = 0.081). Difficult airway and aspiration risk assessment was completed in all cases in both cycles (101 (100%) vs. 203 (100%)).

Substantial improvements were observed during the Before Skin Incision (Time Out) phase. Team member introductions increased significantly from 22 (21.8%) in Cycle 1 to 86 (42.4%) in Cycle 2 (p < 0.001). Verbal confirmation of patient name, procedure, and incision site improved from 61 (60.4%) to 152 (74.9%) (p = 0.010). Antibiotic prophylaxis administered within 60 minutes increased from 67 (66.3%) to 157 (77.3%) (p = 0.041). Discussion of anticipated critical or non-routine steps rose from 18 (17.8%) to 66 (32.5%) (p = 0.006), while anticipated case duration discussion increased markedly from 10 (9.9%) to 86 (42.4%) (p < 0.001). Essential imaging display improved from 18 (17.8%) to 100 (49.3%) (p < 0.001). Nursing confirmation of sterility demonstrated the largest improvement, increasing from 25 (24.8%) to 184 (90.6%) (p < 0.001). Discussion of equipment issues also improved from 8 (7.9%) to 54 (26.6%) (p < 0.001). In contrast, anticipated blood loss discussion declined significantly from 91 (90.1%) to 74 (36.5%) (p < 0.001).

In the Before Patient Leaves the Operating Room (Sign Out) phase, procedure name confirmation improved from 51 (50.5%) in Cycle 1 to 142 (70.0%) in Cycle 2 (p = 0.001). Completion of instrument, sponge, and needle counts increased from 41 (40.6%) to 132 (65.0%) (p < 0.001). Specimen labeling confirmation improved from 28 (27.7%) to 96 (47.3%) (p = 0.001), while documentation of equipment problems addressed rose from 12 (11.9%) to 77 (37.9%) (p < 0.001). The proportion of cases in which none of the Sign Out items were completed decreased significantly from 29 (28.7%) in Cycle 1 to 29 (14.3%) in Cycle 2 (p = 0.003) (Table 1).

**Table 1:** Compliance with WHO SSC parameters before and after intervention at Prince Osman Digna Hospital This table presents compliance with individual components of the WHO SSC across two audit cycles at Prince Osman Digna Hospital. Audit Cycle 1 (n = 101) represents the baseline retrospective assessment, and Audit Cycle 2 (n = 203) represents the prospective assessment following targeted educational and system-based interventions. Data are shown as the frequency (percentage) of cases in which each checklist parameter was completed. Checklist items are grouped according to the three WHO SSC phases: Sign In (before induction of anesthesia), Time Out (before skin incision), and Sign Out (before the patient leaves the operating room). SSC, Surgical Safety Checklist; WHO, World Health Organization

Section	Parameter	First cycle (n = 101)	Second cycle (n = 203)	Improvement (%)	χ² value	p-value
Before Induction	Patient identity, site, procedure, and consent confirmed	89 (88.1%)	186 (91.6%)	+3.5	0.96	0.327
Before Induction	Surgical site marked	83 (82.2%)	161 (79.3%)	-2.9	0.35	0.554
Before Induction	Anesthesia machine and medication check completed	100 (99.0%)	200 (98.5%)	-0.5	0.12	0.725
Before Induction	Pulse oximeter on patient and functioning	98 (97.0%)	199 (98.0%)	+1.0	0.25	0.62
Before Induction	Known allergy documented	16 (15.8%)	45 (22.2%)	+6.4	1.75	0.186
Before Induction	Difficult airway/aspiration risk assessed	101 (100%)	203 (100%)	0.0	-	-
Before Induction	Risk of >500 mL blood loss assessed and managed	10 (9.9%)	35 (17.4%)	+7.5	3.05	0.081
Time Out	Team members introduced by name and role	22 (21.8%)	86 (42.4%)	+20.6	12.74	<0.001
Time Out	Patient name, procedure, and incision site verbally confirmed	61 (60.4%)	152 (74.9%)	+14.5	6.64	0.01
Time Out	Antibiotic prophylaxis administered within 60 minutes	67 (66.3%)	157 (77.3%)	+11.0	4.19	0.041
Time Out	Anticipated critical or non-routine steps discussed	18 (17.8%)	66 (32.5%)	+14.7	7.46	0.006
Time Out	Anticipated case duration discussed	10 (9.9%)	86 (42.4%)	+32.5	34.2	<0.001
Time Out	Anticipated blood loss discussed	91 (90.1%)	74 (36.5%)	-53.6	82.1	<0.001
Time Out	Anesthetist-identified patient-specific concerns discussed	12 (11.9%)	58 (28.6%)	+16.7	10.46	0.001
Time Out	Nursing team sterility confirmed	25 (24.8%)	184 (90.6%)	+65.8	140.6	<0.001
Time Out	Nursing team equipment issues or concerns discussed	8 (7.9%)	54 (26.6%)	+18.7	14.34	<0.001
Time Out	Essential imaging displayed	18 (17.8%)	100 (49.3%)	+31.5	26.9	<0.001
Sign Out	Procedure name verbally confirmed	51 (50.5%)	142 (70.0%)	+19.5	10.87	0.001
Sign Out	Instrument, sponge, and needle counts completed	41 (40.6%)	132 (65.0%)	+24.4	15.85	<0.001
Sign Out	Specimen labeling confirmed	28 (27.7%)	96 (47.3%)	+19.6	10.1	0.001
Sign Out	Equipment problems addressed	12 (11.9%)	77 (37.9%)	+26.0	22.7	<0.001
Sign Out	Key recovery and postoperative management concerns discussed	33 (32.7%)	76 (37.4%)	+4.7	0.67	0.413
Sign Out	None of the above	29 (28.7%)	29 (14.3%)	-14.4	8.9	0.003

## Discussion

Following the adoption of focused, low-cost interventions, this quality improvement project demonstrated significant gains in adherence to the WHO SSC across all three perioperative phases. Even in resource-limited hospital settings, these improvements suggest that structured strategies, specifically standardized checklist documentation, targeted staff education, and continuous audit feedback, can substantially enhance perioperative safety practices and interdisciplinary collaboration.

Rather than reflecting a fundamental change in surgical procedure, the benefits observed in the second audit cycle appear to result from behavioral reinforcement. Compliance becomes more consistent and integrated into standard practice when surgical teams have a thorough understanding of the SSC’s objectives, expectations, and practical implementation. Feedback systems that increased accountability, workflow standardization that reduced ambiguity in documentation, and staff education all contributed to improved checklist adherence. These findings support the notion that team dynamics and human factors, rather than solely structural changes, are critical to long-term improvements in surgical safety.

The audit’s findings align with global research on SSC implementation. In a tertiary care hospital, Krstulović et al. reported significant improvements in checklist compliance after organizational and educational interventions, highlighting the importance of integrating safety procedures into routine clinical workflows [12]. Similarly, Alsadun et al. demonstrated that regular SSC use enhances team engagement and trust in perioperative safety practices, in addition to improving compliance [4]. The current results extend these observations by showing that similar gains can be achieved in a teaching hospital in Sudan through cost-effective, context-appropriate strategies.

Communication-focused components in the Time Out and Sign Out phases, such as team introductions, discussion of anticipated critical events, surgical count confirmation, specimen labeling, and postoperative care planning, showed marked improvement. These gains illustrate the SSC’s role as a tool for teamwork and communication rather than merely a documentation checklist. Enhanced recording of operating team members, patient identifiers, and operative information also demonstrates adherence to the RCS’s guidelines for Good Surgical Practice [1,2]. Comparable behavior-driven improvements have been documented in audits conducted in Lahore, Pakistan, and Bangalore, India, where the use of standardized documentation templates and structured teaching led to better compliance with surgical safety requirements [6,7].

Prior quality improvement initiatives in Sudan similarly reported gains in SSC compliance and operative documentation following audit-based interventions at Dongola, Port Sudan, and Elobeid Teaching Hospitals [8-11]. Building on this growing body of regional evidence, the current project demonstrates the feasibility and effectiveness of audit-and-feedback approaches for strengthening surgical safety culture in low-resource settings.

The SSC has been shown to promote broader cultural change within operating room teams, in addition to checklist compliance. Haynes et al. reported that SSC use across diverse healthcare systems was associated with reduced perioperative morbidity and mortality and improved perceptions of communication and teamwork [13]. Brima et al. emphasized that regular checklist use fosters shared responsibility, reduces hierarchical barriers that can compromise patient safety, and empowers junior staff to raise concerns [14]. In resource-limited, high-workload environments, where structured communication mechanisms can mitigate systemic risks, these cultural benefits are particularly important [13,14].

Despite generally positive results, improvement in a few checklist items, most notably surgical site marking, was limited or inconsistent. This suggests that when informal verification is already practiced, or baseline compliance is high, some steps may be undervalued. Closing these gaps will require continued reinforcement, close monitoring, and ongoing audit cycles to embed checklist practices into routine clinical behavior.

Limitations

This study has several limitations. First, the audit assessed documentation of SSC elements rather than direct intraoperative behavior, meaning it cannot definitively confirm that every checklist step was verbally performed. Second, the possibility of a Hawthorne effect cannot be excluded, as staff awareness of ongoing auditing may have temporarily influenced compliance. Third, conclusions about the long-term sustainability of improvements are limited by the relatively short post-intervention follow-up; this will be addressed in a planned subsequent audit cycle. Finally, as a single-center project, local contextual factors such as staffing patterns and leadership engagement may influence generalizability. Nonetheless, the initiative demonstrates a scalable, low-cost, and effective approach to improving surgical safety documentation and SSC adherence in comparable low-resource healthcare settings.

## Conclusions

This quality improvement project demonstrated that simple, low-cost interventions, particularly staff education, standardized documentation, and regular audit feedback, significantly improved adherence to the WHO SSC. Improvements were most pronounced in communication-focused elements of the Time Out and Sign Out phases, reflecting enhanced teamwork and shared responsibility within the operating room.

Although some checklist components remain inconsistently applied, the overall findings highlight the effectiveness of audit-driven strategies in strengthening surgical safety practices. Continued reinforcement and repeated audit cycles are essential to sustain these gains. This project provides a practical, scalable model, combining education, standardized tools, and audit feedback, for enhancing perioperative safety in similar resource-limited healthcare settings.

## References

[1] (2025). Good Surgical Practice: The Royal College of Surgeons of England. https://www.rcseng.ac.uk/-/media/files/rcs/standards-and-research/gsp/gsp-2014-web.pdf.

[2] (2025). Good Surgical Practice. https://www.rcseng.ac.uk/-/media/Files/RCS/Standards-and-research/GSP/Good-Surgical-Practice-Guide-2025.pdf.

[3] (2009). Implementation manual WHO surgical safety checklist 2009. https://www.who.int/publications/i/item/9789241598590.

[4] Alsadun D, Arishi H, Alhaqbani A (2021). Do we feel safe about the surgical safety checklist? A cross-sectional study between two periods. Glob J Qual Saf Healthc.

[5] Aveling EL, McCulloch P, Dixon-Woods M (2013). A qualitative study comparing experiences of the surgical safety checklist in hospitals in high-income and low-income countries. BMJ Open.

[6] Paul S, Govindaraj R, Govindaraj S (2023). Surgical operative notes compliance with the standard guidelines of the Royal College of Surgeons: a retrospective clinical audit at a tertiary hospital in Bangalore, India. Int J Anat Radiol Surg.

[7] Toru HK, Aizaz M, Orakzai AA, Jan ZU, Khattak AA, Ahmad D (2023). Improving the quality of general surgical operation notes according to the Royal College of Surgeons (RCS) guidelines: a closed-loop audit. Cureus.

[8] Elhadi Bakheet O, Muhammed A, Mohamed A (2024). Evaluation and improvement of the quality of surgical operative notes in the department of general surgery at Dongola Teaching Hospital, Sudan. Cureus.

[9] Abdelbagi AY, Muhammed A, Elamin Elnour MA (2024). Evaluating and improving the quality of surgical operative notes at the Port Sudan Teaching Hospital. Cureus.

[10] Muhammed A, Ibrahim Mohamed AM, Awad Ibrahim AF (2025). Evaluating and improving the quality of surgical operative notes at Elobeid Teaching Hospital: a two-phase audit. Cureus.

[11] Mohamed A, Abdalla M (2024). A study in Wad Madani, Sudan: are we documenting operation notes effectively?. Cureus.

[12] Krstulović J, Hrgović Z, Krešo A, Tavra A, Znaor L, Marušić A (2025). Interventions to improve compliance to surgical safety checklist use: before-and-after study at a tertiary public hospital in Croatia. Healthcare (Basel).

[13] Haynes AB, Weiser TG, Berry WR (2009). A surgical safety checklist to reduce morbidity and mortality in a global population. N Engl J Med.

[14] Brima N, Morhason-Bello IO, Charles V, Davies J, Leather AJ (2022). Improving quality of surgical and anaesthesia care in sub-Saharan Africa: a systematic review of hospital-based quality improvement interventions. BMJ Open.

